# Anti-Inflammatory Effects of *Ribes diacanthum* Pall Mediated via Regulation of Nrf2/HO-1 and NF-κB Signaling Pathways in LPS-Stimulated RAW 264.7 Macrophages and a TPA-Induced Dermatitis Animal Model

**DOI:** 10.3390/antiox9070622

**Published:** 2020-07-15

**Authors:** Na Yeon Kim, Sun Hee Cheong, Kun Jong Lee, Dai-Eun Sok, Mee Ree Kim

**Affiliations:** 1 Department of Food and Nutrition, Chungnam National University, 99 Daehak-ro, Yuseong-gu, Daejeon 34134, Korea; onlyckyou@hanmail.net; 2 Department of Marine Bio Food Science, College of Fisheries and Ocean Science, Chonnam National University, Yeosu 550-749, Korea; sunny3843@jnu.ac.kr; 3 Department of Food and Nutrition, Soongui Women’s College, 10 Soparo 2-gil, Joong Gu, Seoul 100-751, Korea; kunjong@hanmail.net; 4 College of Pharmacy, Chungnam National University, 99 Daehak-ro, Yuseong-gu, Daejeon 34134, Korea; daesok@cnu.ac.kr

**Keywords:** anti-inflammation, HO-1, NF-κB, Nrf2, RAW 264.7 macrophages, *Ribes diacanthum* Pall

## Abstract

*Ribes diacanthum* Pall (RDP) is a Mongolian traditional medicine used to treat renal inflammation. In the present study, we initially investigated the anti-inflammatory effects and mechanisms of action of ethylacetate extract of RDP (EARDP) in RAW 264.7 macrophages stimulated by lipopolysaccharide (LPS) and 12-O-tetradecanoylphorbol-13-acetate (TPA)-induced dermatitis in mice. We demonstrated that EARDP protected against LPS-induced cell death by inhibiting intracellular reactive oxygen species (ROS) and malondialdehyde (MDA) production, as well as the synthesis of pro-inflammatory mediators and cytokines, such as nitric oxide (NO), tumor necrosis factor-α (TNF-α), interleukin-6 (IL-6), and IL-1β. EARDP inhibited the phosphorylation and degradation of inhibitory κB-α (IκB-α) and the activation of nuclear factor (NF)-κB, indicating that the anti-inflammatory effect of EARDP was mediated via the suppression of NF-κB nuclear translocation. In addition, EARDP induced the heme oxygenase-1 (HO-1) expression and nuclear translocation of nuclear factor-E2-related factor 2 (Nrf2), indicating that EARDP induced HO-1 via the Nrf2 pathway in RAW 264.7 cells. Furthermore, EARDP significantly suppressed the protein expression of inducible nitric oxide synthase (iNOS) and cyclooxygenase-2 (COX-2) in LPS-stimulated RAW 264.7 macrophages. However, ZnPP, a specific inhibitor of HO-1, reversed the EARDP-mediated inhibition of NO and TNF-α production in LPS-stimulated RAW 264.7 macrophages. EARDP blocked the phosphorylation of mitogen-activated protein kinase (MAPK) and Akt in LPS-stimulated RAW 264.7 cells. In the in vivo animal model, EARDP significantly and dose-dependently reduced TPA-induced secretion of TNF-α and IL-6 in mouse ear. Based on these results, EARDP represents a promising natural compound, protective against oxidative stress and inflammatory diseases.

## 1. Introduction

Inflammation is an important mechanism contributing to the host defense against pathogenic challenges and the restoration of normal tissue structure [1]. Macrophages are the main pro-inflammatory cells, and they protect the body from external intruders, by releasing pro-inflammatory mediators and cytokines such as nitric oxide (NO), inducible nitric oxide synthase (iNOS) and cyclooxygenase-2 (COX-2), tumor necrosis factor-α (TNF-α), and IL-1β [2]. Under inflammatory conditions, these pro-inflammatory enzymes and cytokines induce cell and tissue injury involved in chronic inflammatory disorders such as hepatitis, rheumatoid arthritis, and atherosclerosis [3,4]. NF-κB regulates the gene expression of several pro-inflammatory cytokines, including TNF-α and IL-1β [5].

Heme oxygenase-1 (HO-1), an inducible rate-limiting enzyme, catalyzes the splitting of free heme into the free iron, carbon monoxide (CO), and biliverdin/bilirubin in heme catabolism. HO-1 and its enzymatic by-products, which mediate the resolution of inflammatory response, are critical regulators of inflammation, predominantly targeting inflammatory cells [2,6]. The anti-inflammatory action of HO-1 is mediated via the suppression of several pro-inflammatory mediators and cytokines [3,4]. Numerous studies also demonstrated that HO-1 and its products, such as CO, suppress the protein expression of iNOS and COX-2, thereby reducing NO and PGE_2_ production [7,8]. The expression of HO-1 is related to several signaling pathways, such as mitogen activated protein kinases (MAPKs), phosphoinositide 3-kinase (PI3K)/protein kinase B (Akt), and the transcription factor nuclear factor-E2-related factor 2 (Nrf2) [9]. MAPKs are common signaling pathways that coordinate the cellular response to a variety of extracellular stimuli. MAPKs play an important role in immune defense, stress response, cellular proliferation, and apoptosis [10]. The MAPK activation modulates the expression of a number of genes including HO-1 via Nrf2 [11]. Nrf2 upregulates the HO-1 gene transcription via cognate DNA binding domains in the HO-1 promoter, leading to the expression of specific inducible proteins, such as HO-1, quinone reductase, and glutathione S-transferase [12]. Accordingly, phytochemicals activate Nrf2 by directly binding to Keap1, resulting in the HO-1 induction [13]. Lipopolysaccharide (LPS), a bacterial endotoxin, triggers the synthesis of pro-inflammatory enzymes and cytokines, such as iNOS, COX-2, ILs, and TNF-α in macrophages [14]. Therefore, reducing LPS-inducible inflammatory mediators and cytokines is regarded as an important strategy to attenuate a variety of inflammation related diseases triggered by macrophages activation.

*Ribes diacanthum* Pall (RDP) is well known as a native Mongolian medicinal plant, the aerial parts of which include leaves, stems, and fruits, which have been traditionally used to treat kidney diseases, bladder infection, and cystitis. In particular, the water extract of RDP is a popular folk medicine used to treat edema and detoxification [15]. Previously, we already found that the extract of RDP (EARDP) containing high amounts of total phenolic and flavonoids showed predominant antioxidant activities [16]. Accordingly, and as expected, EARDP exhibits anti-inflammatory action. Scientific evidence has demonstrated that phenolic compounds and flavonoids present in plants have a number of potential biological properties, such as antioxidants, anti-inflammatory, anticancer, and anti-allergic activities [17,18,19,20]. Recently, polyphenols and antioxidants have been found to exhibit an anti-inflammatory effect via activation of Nrf2 signaling systems [21]. However, the anti-inflammatory action and molecular mechanisms of EARDP have yet to be fully elucidated. In the present study, therefore, we investigated the preventive effects of EARDP, and its possible molecular mechanisms against oxidative stress and inflammation, in both RAW 264.7 macrophages stimulated by LPS and the 12-O-tetradecanoylphorbol-13-acetate (TPA)-induced dermatitis mouse model.

## 2. Materials and Methods

### 2.1. Reagents

Dulbecco’s modified Eagle’s minimum essential medium (DMEM), antibiotics (penicillin and streptomycin), and fetal bovine serum (FBS) were purchased from Gibco Life Technologies (Grand Island, NY, USA). Ez-cytox (EZ-3000) and CytoTox 96 cytotoxicity assay kits were purchased from Promega (Madison, WI, USA). Glutathione (DIGT-250) and a glutathione peroxidase assay kit (EGPX-100) were purchased from Bioassay Systems (Hayward, CA, USA). Antibodies to inhibitory κB-α (IκB-α), iNOS, COX-2, HO-1, Nrf2, p38 kinases (p38), extracellular signal-regulated kinases (ERK), c-Jun N-terminal kinases (JNK), Akt, phosphor (p)-IκB-α, (p)-NF-κB, (p)-ERK, (p)-JNK, (p)-p38, (p)-Akt and actin were purchased from Cell Signaling Technology Inc. (Beverly, MA, USA). The ECL detection agents were purchased from iNtRON Biotechnology, Inc. (Seongnam, Korea). PVDF membranes were purchased from Bio-Rad Laboratories (Alfred Nobel Drive, Hercules, CA). The enzyme-linked immunosorbent assay (ELISA) kits for interleukin-6 (IL-6), IL-1β, and TNF-α were purchased from BD Science, Inc. (San Diego, CA, USA). LPS, Tween-20, DMSO, acetic acid and all other chemicals were purchased from Sigma Aldrich Co. (St. Louis, MO, USA).

### 2.2. Preparation of EARDP

The aerial parts, containing fruits, leaves, and stems, were obtained from fresh RDP plants in the Khentii Mountains of Mongolia. The specimens of RDP were authenticated and kept in the Botany Herbarium of the Department of Biology, Ulaanbaatar University of Mongolia [16]. The dried RDP (3 kg) was extracted in 80% methanol (6 L × 3) at room temperature for 72 h, followed by filtration with Whatman No.2 filter paper (Whatman International Limited, Kent, England, United Kingdom) and evaporation, using a rotary evaporator (N11, Yamato Co., Tokyo, Japan) under reduced pressure. The concentrated 80% MeOH extract (261 g; percentage yield, 8.70%) was dissolved in distilled water. Consecutively, the solution was partitioned in a separating funnel, with an equivalent amount of ethyl acetate (EtOAc). The layer of EtOAc was evaporated to yield 41 g (percentage yield, 15.71%), under reduced pressure, at a temperature lower than 40 °C. The total phenolic and flavonoid contents of EARDP were 0.488 mg/mL and 0.995 mg/mL, respectively [16]. The EARDP contained six polyphenolic substances including protocatechuic acid (121.96 mg/100g), epicatechin (92.31 mg/100g), catechol (30.86 mg/100g), syringic acid (19.14 mg/100g), gallic acid (6.28 mg/100g), and 4-methylcatechol (4.55 mg/100g). The EARDP were stored at −20°C for further experiments.

### 2.3. Cell Culture, Cell Viability, and Cytotoxicity Assays

Murine RAW 264.7 macrophages were obtained from the Korean Cell Line Bank (Seoul, Korea). Cells were maintained in DMEM containing 10% (*v/v*) heat-inactivated FBS, glutamine (1 mM), streptomycin (50 μg/mL), and penicillin (100 U/mL) at 37 °C, in a humidified atmosphere containing 5% CO_2_. The effect of EARDP treatment on cell viability was evaluated via a water-soluble tetrazolium (WST) assay. For the determination of cell viability, cells were pre-incubated in a 96-well plate (1.0 × 10^5^ cells/well) with EARDP (0–125 µg/mL), for 24 h and stimulated by LPS (2 µg/mL) for 24 h, followed by the addition of 10 μL WST solution to the cell suspension (1 × 10^5^ cells/mL per well of the 96-well plates), and further incubation of the mixture for 1 h at 37 °C. The formazan crystal in cells was dissolved in DMSO and quantified by measuring the absorbance at 450 nm using a microplate reader (Labsystem, Multiskan, Helsinki, Finland). In the control cells, the optical density of the formazan was regarded as 100% cell viability. Cytotoxicity was quantitatively determined by measuring the activity of released lactate dehydrogenase (LDH) from the cytoplasm into the culture medium of damaged cells. Cells were plated on a 96-well plate (1.0 × 10^5^ cells/well) and incubated with EARDP for 24 h, and the absorbance at 490 nm was read with a background control as the blank.

### 2.4. Determination of NO Production and Pro-Inflammatory Cytokines (TNF-α, IL-6, and IL-1β) Assays

The NO levels were determined from the nitrite, which is a stable end product of NO oxidation, using a method based on the Griess reaction. A 50 µL aliquot of each supernatant was mixed with 50 µL of Griess reagent (0.1% naphthylethylenediamine dihydrochloride and 1% sulfanilamide in 2.5% phosphoric acid), and then the absorbance was determined at 525 nm using an ELISA plate reader. In the culture medium, the concentrations of TNF-α, IL-6, and IL-1β were determined using ELISA kits (BD Science, San Diego, CA, USA).

### 2.5. Measurement of Intracellular Glutathione (GSH), ROS, and MDA Levels

For the determination of intracellular GSH, reactive oxygen species (ROS), and malondialdehyde (MDA) levels, the cells (1.0 × 10^5^ cells/well) were treated with different concentrations of EARDP (25, 50, and 75 μg/mL) for 22 h and stimulated by LPS (2 µg/mL) for 2 h. The intracellular GSH levels were measured using the method described previously [22]. The GSH level in the supernatants separated from cell homogenates were analyzed using a GSH assay kit purchased from BioAssay Systems (Hayward, CA, USA), according to the manufacturer’s instructions. The intracellular ROS level was measured via a fluorophore 2,7-dichlorofluorescein diacetate (DCFH-DA) assay [23]. After incubating the cells in a 96-well plate for 24 h, the cells were washed twice with Hank’s balanced salt solution, then incubated with 100 μM DCFH-DA at 37 °C for 50 min. Finally, the fluorescence intensity of DCF was recorded, with an excitation wavelength of 485 nm and an emission wavelength of 535 nm, using a fluorometer (Beckman Coulter, CA, USA). For the determination of cellular lipid peroxidation, the intracellular MDA level was determined using an MDA assay kit (Sigma Aldrich Co., St. Louis, MO, USA).

### 2.6. Preparation of Nuclear and Cytosolic Extraction

Nuclear and cytosolic extractions were fractionated using a procedure reported previously [24] with a Nuclear Extraction Kit supplied by Cayman Chemical Company, Inc. (Ann Arbor, MI, USA), according to the manufacturer’s instructions. Briefly, RAW 264.7 macrophages (1.0 × 10^7^ cells/well) were harvested and pelleted via centrifugation at 300× g for 5 min at 4 °C. The cell pellets were added to 500 µL hypotonic buffer, and then incubated on ice for 15 min. The cells were mixed with 100 µL of 10% Nonidet P-40 Assay Reagent. Nuclei were extracted via centrifugation at 14,000× g for 30 s at 4 °C, and the supernatant was stored as cytoplasmic extract at −80 °C until use. The nuclei were extracted with nuclear extraction buffer containing protease inhibitors and phosphatase inhibitors for 30 min on ice. Insoluble precipitate was removed via centrifugation at 14,000× g for 10 min at 4 °C. Finally, the supernatant was used as a nuclear extract.

### 2.7. Western Blotting Analysis

RAW 264.7 macrophages (1 × 10^6^) were harvested and pelleted via centrifugation at 14,000× g for 3 min at 4 °C, and then washed once with cold phosphate buffer saline. The pelleted cells were resuspended in a lysis buffer (5 mM EDTA, 0.1% Triton X-100, 50 mM Tris-HCl pH 7.4, 150 mM NaCl, protease and phosphatase inhibitor cocktail) and centrifuged at 14,000× g for 10 min at 4 °C. Protein expression of iNOS, COX-2, HO-1, Nrf2, IκBα, NF-κB, MAPKs, and Akt or corresponding phosphorylated forms was determined using a Western blot analysis. The cellular proteins were collected using the PRO-PREP protein extraction kit (iNtRON Biotechnology, Inc., Seongnam, Korea) and the protein concentration of the cell lysates was measured using a SMART™ BCA protein assay kit (iNtRON Biotechnology, Inc., Seongnam, Korea), according to the manufacturer’s instructions. An equal quantity of protein from each sample (30 µg) was subjected to electrophoresis on 7.5%, 10% or 12.5% sodium dodecyl sulfate-polyacrylamide gels, and then electrophoretically transferred onto polyvinylidene fluoride membranes (Bio-Rad, Hercules, CA, USA). The membrane was incubated with 5% skim milk in TBS buffer (20 mM Tris–HCl containing 150 mM NaCl, pH 7.4) for 2 h, to block the non-specific binding of antibodies. The membrane was sequentially incubated with different primary antibodies, horseradish peroxidase-conjugated secondary antibodies for 1 h, and then washed five times with TBS-T (TBS containing 0.05% Tween-20). The immunoreactive bands were visualized with the WEST One^™^ western blot detection system (iNtRON Biotechnology, Inc., Seongnam, Korea). The relative density of the protein expression was measured using ImageJ software (version 1.50i for Windows; NIH, USA).

### 2.8. In Vivo Animal Experiment

Male ICR mice (6 weeks, 25–28 g) were obtained from Animal Husbandry of Damul Science (Daejeon, Korea). The mice were kept in cages (3 or 4 mice per cage) under a specific pathogen-free condition (23 ± 2 °C and 55 ± 10% relative humidity), with a 12 h light/dark cycle (lights on 06:00–18:00 h), and allowed free access to water and a pelleted commercial diet (Samyang Co., Seoul, Korea) ad libitum. All experiments were conducted in accordance with the NIH Guide for the Care and Use of Laboratory Animals (NIH Publication No. 85-23, 1985, revised 1996), and were approved by the Institutional Animal Care and Use Committee of Chungnam National University (Registration NO. CNU-00037). After 1 week of acclimation, the mice were randomly divided into five treatment groups of 7 mice each, and then allowed free access to water and treated with EARDP (200 μL, 25, 50 or 100 mg/kg, per orally) for 10 days. Edema of the ear was induced on the right ear via topical application of 1 μg/ear of TPA dissolved in 10 μL of acetone. The ear thickness was measured using a Digimatic Micrometer (Mitsutoyo Co., Tokyo, Japan) before TPA application. After 24 h, the edema was expressed as the increase in ear thickness due to skin inflammation. The ear tissues were frozen in liquid nitrogen, and homogenized in 1 mL of ice-cold lysis buffer, containing 150 mM NaCl, 5 mM EDTA, 0.1% Triton X-100, 50 mM Tris-HCl (pH 7.4), and phosphatase inhibitor cocktails. The lysates were centrifuged at 14,000× g for 15 min at 4 °C, and then stored at −80 °C until use.

### 2.9. Statistical Analysis

The data were expressed as the mean ± SEM, and *n* refers to the number of sample replicates. All statistical analyses were carried out using the SPSS version 12.0 (Statistical Package for Social, SPSS Inc., Chicago IL, USA) software. The data were evaluated by one-way analysis of variance (ANOVA) followed by Duncan’s multiple range test or Tukey’s multiple comparison tests. Differences at the * *p* < 0.05 were considered statistically significant.

## 3. Results

### 3.1. Cytotoxicity of EARDP on RAW 264.7 Macrophages

**Figure 1:**
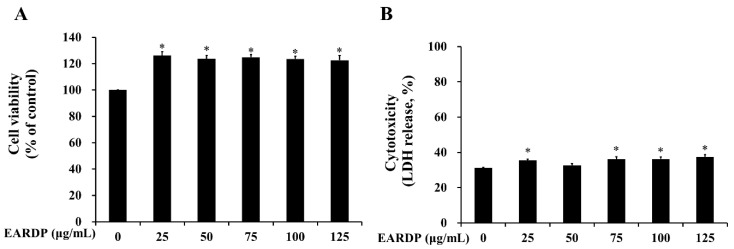
Effect of extract of *Ribes diacanthum* Pall (EARDP) on the cell viability of RAW 264.7 macrophage cells. (**A**) Cells were seeded on a 96-well plate and treated with varying concentrations of EARDP (25–125 μg/mL) for 24 h for WST assay. (**B**) LDH assay of RAW 264.7 macrophages treated with EARDP (25–125 μg/mL) for 24 h. Data are expressed as mean ± SEM values (*n* = 4 independent experiments). * *p* < 0.05 vs. vehicle control.

In the present study, WST and LDH cytotoxicity assays were utilized to measure the cytotoxicity of EARDP against RAW 264.7 macrophages. As shown in Figure 1A, cell viability was not significantly altered after the incubation of RAW 264.7 macrophages with EARDP concentrations up to 125 μg/mL. To further investigate the cytotoxic effects, cells were treated with varying concentrations of EARDP (25–125 μg/mL) for 24 h, to detect the release of LDH activity, an indicator of cellular damage and cytotoxicity, from RAW 264.7 macrophages. Figure 1B showed that cells were not significantly damaged by treatment with EARDP (25–125 μg/mL). These results suggested that EARDP concentrations up to 125 μg/mL enhanced the cell viability slightly by approximately 20% but did not lead to cytotoxicity in RAW 264.7 macrophages. A previous study also demonstrated that *Sanguisorba officinalis* ethanol extract (50–100 μg/mL) showed increased values of cell viability, NO, and PGE_2_ in RAW264.7 cells and peritoneal macrophages, maybe due to some immunostimulatory substances in the extract from medicinal plant [25]. Therefore, the subsequent experiments were conducted at three different concentrations, ranging between 25 and 75 μg/mL (25, 50 and 75 μg/mL) of EARDP.

### 3.2. Effect of EARDP on Intracellular GSH Level, ROS Generation, and Lipid Peroxidation in LPS-Stimulated RAW 264.7 Macrophages

**Figure 2:**
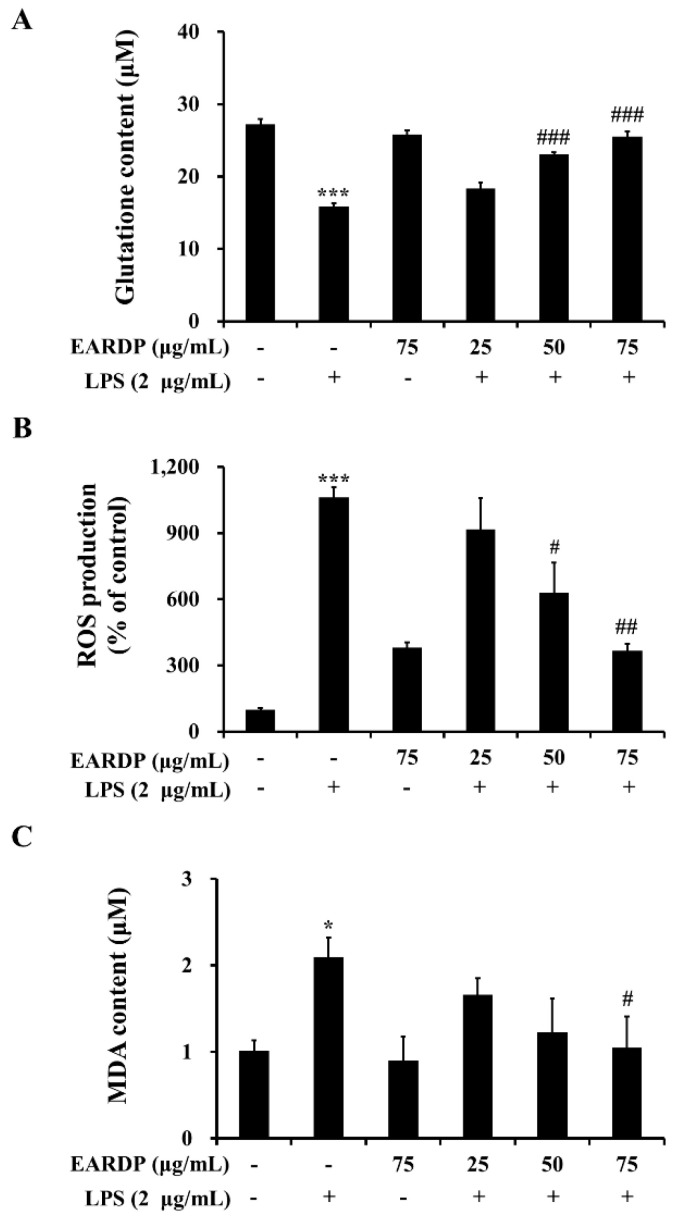
Effects of EARDP on (**A**) intracellular GSH content, (**B**) ROS production, and (**C**) MDA content of LPS-stimulated RAW 264.7 macrophages. Cells were pretreated with EARDP (25, 50, and 75 μg/mL) for 22 h, followed by incubation for 2 h with LPS (2 µg/mL). Data are expressed as mean ± SEM values of experiments in triplicate (*n* = 3). * *p* < 0.05, *** *p* < 0.001 vs. vehicle control; ^#^ *p* < 0.05, ^##^ *p* < 0.01, ^###^ *p* < 0.001 vs. only LPS-treated cells.

The present study investigated whether EARDP affected intracellular GSH level and LPS-induced ROS generation and lipid peroxidation in RAW 264.7 macrophages. After RAW 264.7 cells were treated with LPS for 2 h, a decreased level of intracellular GSH was observed in RAW 264.7 cells. However, pretreatment with EARDP at different concentrations significantly increased intracellular GSH levels from 18.33 μM to 25.56 μM in a dose-dependent manner (Figure 2A). In addition, treatment with LPS markedly induced ROS generation compared with untreated cells. However, pretreatment with EARDP at the concentration of 50 and 75 μg/mL effectively suppressed LPS-induced ROS generation in RAW 264.7 cells (Figure 2B). Meanwhile, MDA is a low-molecular-weight end product released during the decomposition of lipid peroxidation products [26]. As shown in Figure 2C, treatment with LPS for 2 h doubled the MDA level compared with that of untreated cells, whereas pretreatment with 75 μg/mL EARDP significantly decreased LPS-induced MDA levels. These results demonstrate that EARDP has protective effects against oxidative cell damage induced by LPS via the suppression of intracellular ROS and MDA levels in RAW 264.7 macrophages.

### 3.3. Effects of EARDP on Morphological Changes, Production of Nitrite, TNF-α, IL-6 and IL-1β in LPS-Stimulated RAW 264.7 Macrophages

**Figure 3:**
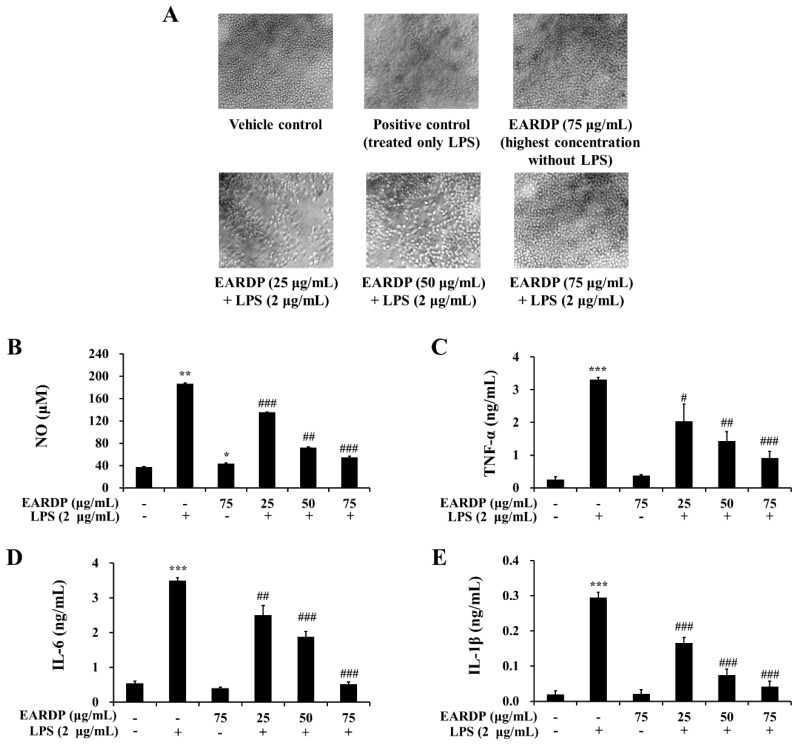
Effects of EARDP on (**A**) morphological changes, production of (**B**) NO, (**C**) TNF-α, (**D**) IL-6, and (**E**) IL-1β of RAW 264.7 macrophages activated with LPS. The cells were simultaneously exposed to EARDP (25, 50, and 75 μg/mL), with or without LPS (2 μg/mL) for 24 h and stimulated by LPS (2 µg/mL) for 24 h. The levels of NO, TNF-α, IL-6, and IL-1β were determined, as described in the Materials and Methods section. Data are expressed as mean ± SEM values of experiments in triplicate (*n* = 3). * *p* < 0.05, *** *p* < 0.001 vs. vehicle control; ^#^ *p* < 0.05, ^##^ *p* < 0.01, ^###^ *p* < 0.001 vs. only LPS-treated cells.

To determine the cytotoxic potential of EARDP, its effect on morphological changes in RAW 264.7 macrophages exposed to LPS was examined (Figure 3A). Compared with the vehicle control, an apparent morphological change was observed in RAW 264.7 macrophages exposed to LPS for 24 h. Meanwhile, EARDP dose-dependently increased the viability of LPS-treated cells. Especially, the treatment with 75 μg/mL of EARDP completely blocked the effect on LPS-treated cells, similar with 75 μg/mL of EARDP untreated with LPS. At the non-cytotoxic concentrations (25–75 μg/mL), the anti-inflammatory effect of EARDP in LPS-stimulated RAW 264.7 cells was assessed via the estimation of the synthesis of NO, TNF-α, IL-6 and IL-1β. As shown in Figure 3B, EARDP dose-dependently inhibited the generation of NO in LPS-stimulated RAW 264.7 macrophages. In order to evaluate the potential effect of EARDP on the generation of several pro-inflammatory cytokines, including TNF-α, IL-6 and IL-1β, RAW 264.7 macrophages were activated with LPS for 24 h, in the absence or presence of non-cytotoxic concentrations of EARDP. We found that EARDP markedly and dose-dependently suppressed the production of TNF-α, IL-6 and IL-1β in LPS-stimulated RAW 264.7 macrophages (Figure 3C–E). These results indicate that EARDP protects against LPS-induced cell death, by inhibiting the synthesis of inflammatory mediators and cytokines.

### 3.4. Effects of EARDP on Expression Levels of IκB-α, p-IκB-α, and p-NF-κB in LPS-Stimulated RAW 264.7 Macrophages

**Figure 4:**
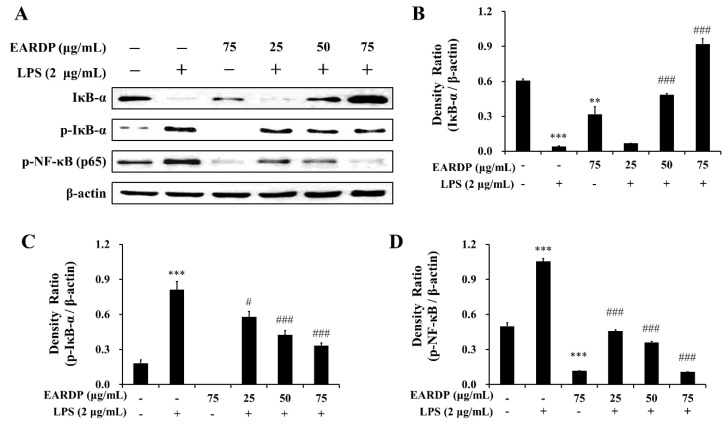
Effects of EARDP on the (**A**) protein expression of IκB-α, p-IκB-α, and p-NF-κB (p65 subunit) in LPS-stimulated RAW 264.7 macrophages. (**B**–**D**) The expression of IκB-α, p-IκB-α, and p-NF-κB (p65 subunit) was quantified by image analysis. Relative densities were calculated as the ratio of IκB-α, p-IκB-α, and p-NF-κB expression to β-actin expression, respectively. Data are expressed as mean ± SEM values of experiments in triplicate (*n* = 3). ** *p* < 0.01, *** *p* < 0.001 vs. vehicle control; ^#^ *p* < 0.05, ^###^ *p* < 0.001 vs. only LPS-treated cells.

Next, we examined the effects of EARDP on the phosphorylation and degradation of IκB-α, which is one of the cellular proteins to inhibit the NF-κB in the cytoplasm. As shown in Figure 4A–C, IκB-α was phosphorylated and degraded after treatment with LPS for 10 min in RAW 264.7 macrophages, but phosphorylation and degradation were markedly and dose-dependently inhibited by treatment with EARDP. To further elucidate the suppressive mechanisms of EARDP on the activation of NF-κB induced by LPS, we examined the effect of EARDP on phosphorylation of the NF-κB p65 subunit in the cytoplasm. As shown in Figure 4A,D, LPS-induced phosphorylation of NF-κB p65 was significantly and dose-dependently inhibited via treatment with EARDP for 24 h. Taken together, these findings suggest that the anti-inflammatory effect of EARDP is mediated via preventing the phosphorylation and degradation of IκB-α and phosphorylation of NF-κB, resulting in the inhibition of the NF-κB signaling pathway in LPS-stimulated RAW 264.7 macrophages.

### 3.5. Effects of EARDP on Nrf2-Mediated HO-1 Induction in RAW 264.7 Macrophages

**Figure 5:**
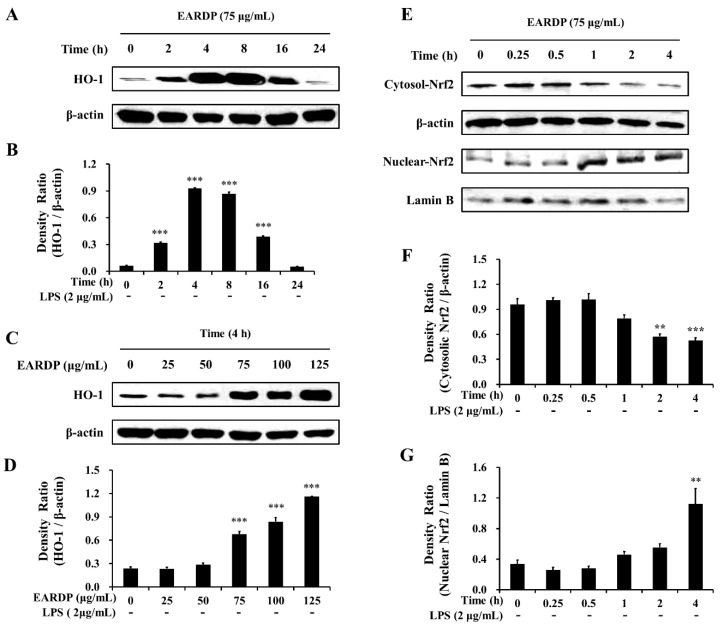
Effects of EARDP on (**A**–**D**) HO-1 protein expression in RAW 264.7 macrophages. (**A**,**B**) Cells were incubated with 75 μg/mL EARDP for the indicated periods. (**C**,**D**) Cells were incubated with the indicated concentrations of EARDP for 4 h. Relative densities were calculated as the ratio of HO-1 expression to β-actin expression. Data are expressed as mean ± SEM values of experiments in triplicate (*n* = 3). *** *p* < 0.001 vs. vehicle control. (**E**) Cells were incubated with 75 μg/mL EARDP for the indicated periods. (**F**,**G**) EARDP-induced Nrf2 degradation in the cytosolic fraction and Nrf2 expression in the nuclear fraction in LPS-unstimulated RAW 264.7 macrophages were quantified by image analysis. Relative densities were calculated as the ratio of cytosolic Nrf2 degradation to β-actin expression, and the ratio of nuclear Nrf2 expression to Lamin B expression, respectively. Data are expressed as mean ± SEM values of experiments in triplicate (*n* = 3). ** *p* < 0.01, *** *p* < 0.001 vs. vehicle control.

In the present study, EARDP significantly promoted HO-1 expression, with the maximum increase detected by 4 h. At this time, HO-1 expression significantly and dose-dependently increased between 75 and 125 μg/mL (Figure 5A–D). We also found that Nrf2 levels gradually decreased in the cytoplasmic fraction, whereas they increased in the nuclear fraction treated with 75 μg/mL EARDP at 4 h (Figure 5E–G), suggesting that EARDP directly affects HO-1 protein expression via the nuclear translocation of Nrf2. These results demonstrated that HO-1 induction by EARDP was associated with the Nrf2 nuclear translocation pathway in RAW 264.7 macrophages.

### 3.6. Effects of EARDP on iNOS, COX-2, and HO-1 Expression in LPS-Stimulated RAW 264.7 Macrophages

**Figure 6:**
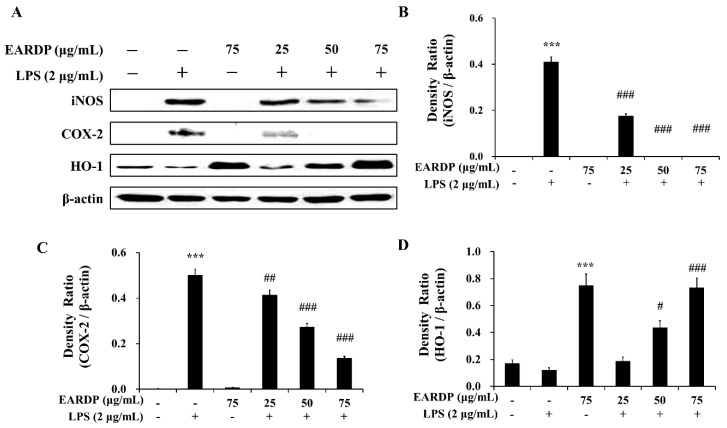
Effects of EARDP on iNOS, COX-2, and HO-1 expression in LPS-stimulated RAW 264.7 macrophages. (**A**) Effects of EARDP on LPS-induced iNOS, COX-2, and HO-1 expression determined by Western blot analysis. (**B**–**D**) The expression of iNOS, COX-2, and HO-1 was quantified by image analysis. Relative densities were calculated as the ratio of iNOS, COX-2, and HO-1 expression to β-actin expression, respectively. Data are expressed as mean ± SEM values of experiments in triplicate (*n* = 3). *** *p* < 0.001 vs. vehicle control; ^#^ *p* < 0.05, ^##^ *p* < 0.01, ^###^ *p* < 0.001 vs. only LPS-treated cells.

We investigated the effects of EARDP on LPS-induced iNOS, COX-2, and HO-1 protein expression in RAW 264.7 macrophages. As shown in Figure 6, LPS-activated RAW 264.7 cells showed an elevated expression of iNOS and COX-2, whereas EARDP significantly and dose dependently attenuated the protein expression of iNOS and COX-2. We also investigated whether EARDP affected the HO-1 expression in LPS-stimulated RAW 264.7 cells. In the present study, EARDP markedly induced HO-1 expression in a dose-dependent manner in LPS-stimulated RAW 264.7 cells, as well as the cells treated with EARDP alone (Figure 6A,D). These results demonstrated that EARDP effectively attenuated the LPS-induced pro-inflammatory enzymes, such as iNOS and COX-2, via Nrf2-mediated HO-1 expression in RAW 264.7 macrophages.

### 3.7. Effects of HO-1 Protein Expression on the Inhibition of NO and TNF-α by EARDP in LPS-Stimulated RAW 264.7 Macrophages

**Figure 7:**
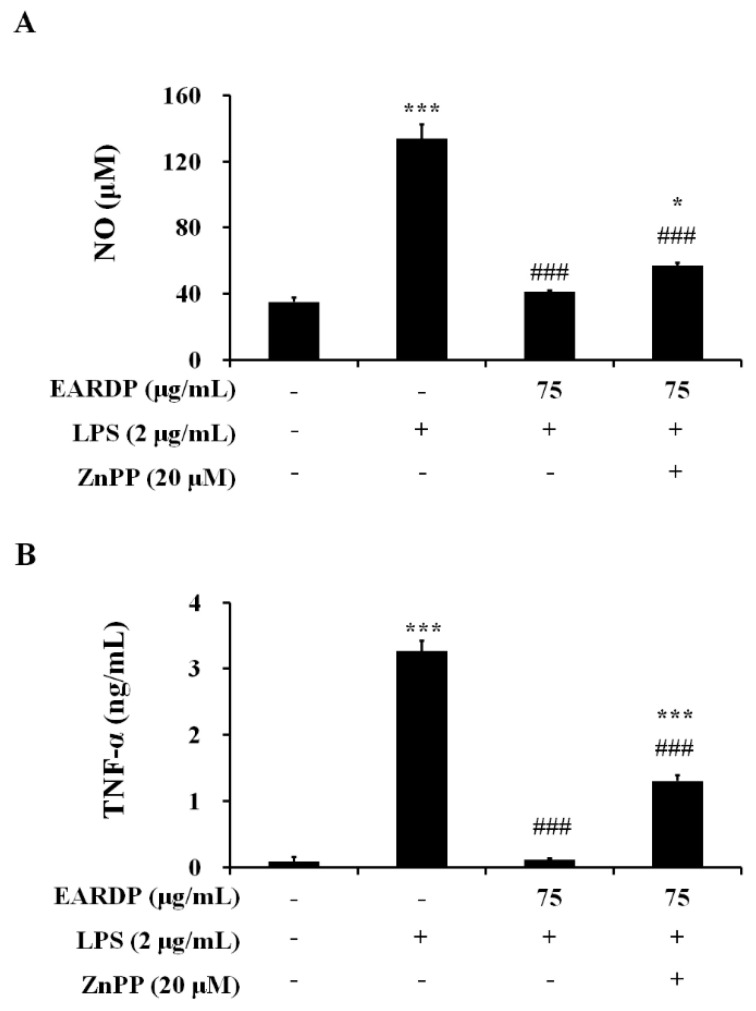
Effects of ZnPP on (**A**) NO, and (**B**) TNF-α production by EARDP in LPS-stimulated RAW 264.7 macrophages. Cells were pre-treated with ZnPP (20 μM) for 2 h, followed by treatment with EARDP (75 μg/mL) and stimulation with LPS (2 μg/mL) for 24 h. Data are expressed as mean ± SEM values of experiments in triplicate (*n* = 3). * *p* < 0.05, *** *p* < 0.0001 vs. vehicle control; ^###^ *p* < 0.001 vs. only LPS-treated cells.

To confirm that the anti-inflammatory activity of EARDP correlated with HO-1 protein induction via the Nrf2 pathway, we analyzed the effects of zinc protoporphyrin IX (ZnPP), a known competitive inhibitor of HO-1. The cells were pretreated with ZnPP (20 µM), and then treated with EARDP (75 µg/mL), followed by stimulation with LPS (2 µg/mL) for 24 h. As shown in Figure 7, pretreatment with ZnPP partially suppressed the inhibitory effects of EARDP on the production of pro-inflammatory mediators, including NO and TNF-α in LPS-stimulated RAW 264.7 cells. These results indicate that Nrf2-mediated HO-1 induction contributes to the anti-inflammatory properties of EARDP on the expression of pro-inflammatory mediators.

### 3.8. Role of the MAPK and PI3K/Akt Pathways in the EARDP-Induced HO-1 Expression

**Figure 8:**
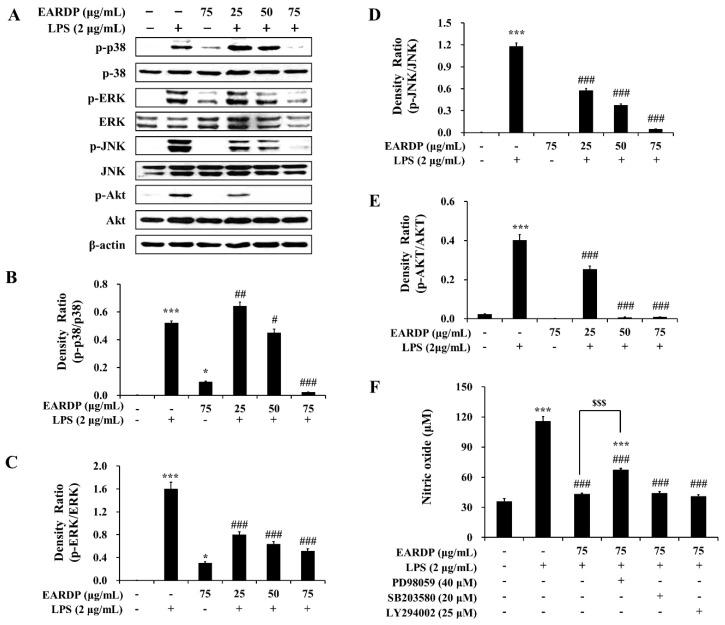
Effects of EARDP on (**A**) the phosphorylation of MAPKs and Akt in LPS-stimulated RAW 264.7 macrophages. (**B**–**E**) The expression of phosphorylated p38, ERK, JNK, and Akt was quantified by image analysis. Data are expressed as mean ± SEM values in triplicate (*n* = 3). * *p* < 0.05, *** *p* < 0.001 vs. vehicle control; ^#^ *p* < 0.05, ^##^ *p* < 0.01, ^###^ *p* < 0.001 vs. only LPS-treated cells. (**F**) Effects of specific inhibitors of MAPKs and Akt on nitrite synthesis by EARDP in LPS-stimulated RAW 264.7 macrophages. Cells were pre-incubated with specific inhibitors of MAPKs and Akt (40 μM PD98059, 20 μM SB203580, 25 μM LY294002) for 1 h, followed by treatment with 75 μg/mL EARDP and 2 μg/mL LPS for 24 h. After 24 h, the concentration of nitrite was determined using the Griess reagent. Data are expressed as mean ± SEM values in triplicate (*n* = 3). *** *p* < 0.001 vs. vehicle control; ^###^ *p* < 0.001 vs. only LPS-treated cells; ^$$$^ *p* < 0.001 vs. LPS with EARDP (75 μg/mL).

**Figure 9:**
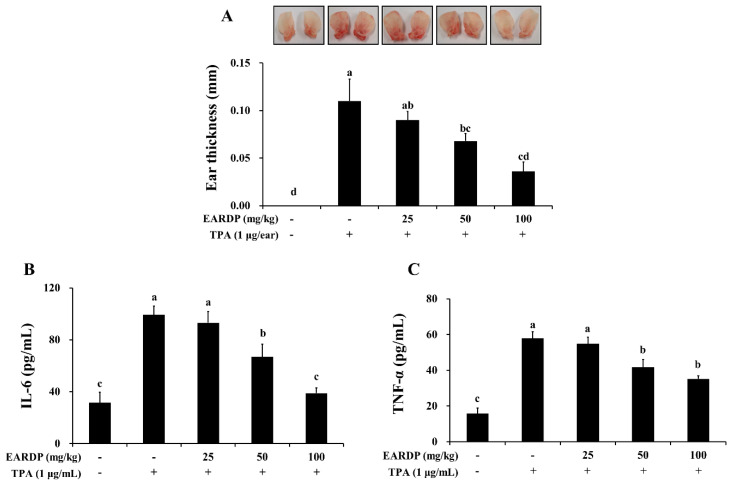
Inhibitory effects of EARDP on TPA-induced dermatitis in mice. The mice were treated with EARDP (200 μL, 25, 50 or 100 mg/kg, p.o.) for 10 days. Edema was induced on the back of right ear via topical application of 1 μg/ear of TPA dissolved in 10 μL of acetone. (**A**) Ear thickness, and the levels of (**B**) TNF-α and (**C**) IL-6 in ear tissues were measured as described in Materials and Methods. Data are expressed as mean ± SEM values (*n* = 7). Different letters on the bars represent significant differences in Duncan’s multiple range test at *p* < 0.05.

In the present study, we examined the role of MAPK and Akt signaling pathways in EARDP-mediated inhibition of LPS-induced inflammatory responses. As shown in Figure 8, LPS treatment substantially promoted the phosphorylation of three MAPKs, such as p38, ERK, and JNK. In contrast, the phosphorylation of three types of MAPKs was significantly and dose-dependently inhibited by treatment with EARDP in LPS-stimulated RAW 264.7 macrophages (Figure 8A–D). In addition, EARDP markedly suppressed the phosphorylation of Akt, which is linked to COX-2 or iNOS expression via the activation of NF-κB in LPS-stimulated RAW 264.7 macrophages (Figure 8A,E). These results suggested that EARDP inhibited LPS-induced MAPK and Akt activation in RAW 264.7 macrophages. Furthermore, we determined the production of nitrite in the presence of specific inhibitors, including ERK inhibitor (PD98059), p38 inhibitor (SB203580), and Akt inhibitor (LY294002), to investigate the involvement of MAPKs pathway in the anti-inflammatory activities of EARDP. As shown in Figure 9F, LPS-induced NO synthesis was markedly suppressed in the presence of EARDP. However, the inhibitory effect of EARDP and specific inhibitors, including PD98059, SB203580, and LY294002 co-treatment on NO production was not greater than that observed with EARDP in LPS-stimulated RAW264.7 macrophages (Figure 8F). Although the inhibitory effects of specific inhibitors on LPS-induced NO production were unclear, our results suggest that EARDP exhibits anti-inflammatory properties, by inhibiting the phosphorylation of MAPKs, including p38, ERK and JNK, and Akt, in LPS-stimulated RAW 264.7 macrophages.

### 3.9. Anti-Inflammatory Effects of EARDP in TPA-Induced Mouse Model

To determine the in vivo anti-inflammatory properties of EARDP, we used a TPA-induced model of mouse ear edema. After topical application of 1 μg TPA in acetone to the ear of a mouse, the average thickness of the ear markedly increased compared to the control group. In contrast, EARDP significantly and dose-dependently inhibited the increases in ear thickness induced by TPA (Figure 9A). We further investigated the effect of EARDP (25, 50 and 100 mg/kg) on inflammatory cytokines, such as IL-6 and TNF-α, in each of the ear punch biopsies by ELISA. EARDP dose-dependently inhibited the formation of TNF-α and IL-6 induced by TPA (Figure 9B,C). These results indicate that EARDP exerts anti-inflammatory effects, by inhibiting the synthesis of inflammatory cytokines, in mice exposed to TPA-induced skin inflammation.

## 4. Discussion

RDP is one of the Mongolian traditional plants belonging to the Saxifragaceae family. As a folk medicine, RDP has been widely used to treat diseases of the urinary system. Recently, a few pharmacological properties of RDP have been reported, including the inhibition of renal fibrosis and nephrotoxicity [15,27,28]. However, the mechanisms underlying the anti-inflammatory activity of EARDP have yet to be reported. In the present study, therefore, we assumed that the EARDP, which contains a large amount of total phenolic and flavonoids, and potent free radical scavenging activities, has anti-inflammatory effects on murine macrophage cells stimulated with LPS, and performed a series of experiments to elucidate the anti-inflammatory mechanisms mediated via Nrf2-mediated HO-1 expression. This is the first study to demonstrate that EARDP has antioxidant and anti-inflammatory effects mediated by HO-1 expression via the activation of Nrf2 and the inhibition of the NF-κB pathway in LPS-induced RAW 264.7 macrophages. Furthermore, we investigated the in vivo anti-inflammatory properties using TPA-induced mouse models of dermatitis.

NO, a free oxygen radical, acts as a cytotoxic agent in the pathophysiology of several inflammatory diseases. In activated macrophages, the production of NO via iNOS may contribute to the pathology of various acute and chronic inflammatory conditions [29]. PGE_2_, which is one of the strongest inflammatory mediators, is transformed from arachidonic acid via COX2 catalytic reaction. The expression of pro-inflammatory enzymes, including iNOS and COX-2, plays a crucial role in activated macrophages, via the synthesis of NO and PGE_2_ [30,31]. In addition, pro-inflammatory cytokines, including TNF-α, IL-6, and IL-1β, play key roles in regulating inflammation and tumor progression [32]. In the present study, we found that EARDP significantly inhibited the production of NO in LPS-stimulated RAW 264.7 cells, with no cytotoxicity. The inhibitory action of EARDP on LPS-induced NO synthesis appears to involve the suppression of iNOS and COX-2 expression. In addition, EARDP significantly suppressed the LPS-induced production of TNF-α, IL-6, and IL-1β in LPS-activated macrophages, in a dose-dependent manner. It has been demonstrated that LPS induces the generation of various ROS, which triggers inflammatory responses and cellular damage [33]. In our present study, we also observed that EARDP markedly altered the levels of markers of oxidative stress, such as intracellular GSH, ROS, and MDA, as well as pro-inflammatory mediators and cytokines in LPS-activated RAW 264.7 cells.

While in an inactivated state, NF-κB heterodimer (p65 and p50) is located in the cytoplasm complexed with the inhibitory protein IκB-α. When the enzyme IκB kinase (IKK) is activated by a variety of extracellular signals, IKK phosphorylates and subsequently degrades the IκB-α, which results in the release of the activated and phosphorylated NF-κB p65 subunit [34,35]. Activated NF-κB translocates to the nucleus from the cytoplasm to bind target DNAs, resulting in the expression of genes encoding downstream inflammatory mediators. Therefore, NF-κB is a major factor regulating the expression of genes associated with pro-inflammatory enzymes and cytokines, including iNOS, COX-2, IL-1β, and TNF-α [35]. Several previous studies demonstrated that a variety of anti-inflammatory agents, such as obovatol, quercitrin gallate, and cordycepin, suppress NF-κB signaling [34,36,37]. Thus, we investigated the inhibitory effects of EARDP on NF-κB activation, a key signal transduction pathway leading to iNOS expression, in LPS-stimulated RAW 264.7 macrophages. Our results clearly demonstrated that LPS induced the phosphorylation and degradation of IκB-α and the phosphorylation of NF-κB was significantly attenuated by the pre-treatment of EARDP in RAW 264.7 cells stimulated with LPS. In the control cells, the degradation of IκB-α and the phosphorylation of NF-κB p65 subunit were affected by the treatment of EARDP alone. Similar to our results, some previous studies indicated that the treatment of extracts from medicinal plants alone stimulated the degradation of IκB-α, NO, PGE_2_ production, iNOS expression, MAPKs and Akt signaling pathways, due to the immunostimulatory components or intrinsic properties; not due to the presence of endotoxins of the extract [25,38]. Therefore, EARDP exerts anti-inflammatory effects by suppressing LPS-induced NO, TNF-α, IL-6, and IL-1β, and inhibits the expression of iNOS, mediated by the inhibition of the NF-κB pathway.

As another possible mechanism, Nrf2 transcription factor is located in the cytoplasm bound to the inhibitory protein Keap1, under normal physiological conditions. Under oxidative stress, however, Nrf2 is released from Keap1, and translocated into the nucleus, where it combines with antioxidant response elements (ARE), to regulate the transcription of antioxidant genes, such as inducible enzyme HO-1 [39,40]. HO-1 acts as an antioxidant, by regulating the levels of ROS, resulting in anti-inflammatory properties [41]. The advantageous effects of Nrf2 mediated HO-1 induction have been attributed to a variety of factors, including the release of CO, the formation of biliverdin/bilirubin, and the degradation of the pro-oxidant heme [42,43]. In particular, the final products of heme catabolism exert antioxidant effects via neutralization of intracellular ROS [44]. Meanwhile, CO suppresses LPS-induced PGE_2_, TNF-α, IL-1β, and NO formation via the inhibition of NF-κB activation in macrophages. Therefore, the up-regulation of HO-1 is believed to reduce the detrimental consequences of excessive inflammation [45,46]. In the present study, we found that EARDP markedly inhibited the expression of iNOS and COX-2, and the synthesis of NO and PGE_2_, by upregulating HO-1 expression via Nrf2 activation in LPS-stimulated RAW 264.7 macrophages. Moreover, treatment with HO-1 inhibitor ZnPP partially reversed the suppressive effects of EARDP on LPS-induced NO and TNF-α production. Similar to our results, previous studies showed that other plant-derived compounds, such as resveratrol, curcumin, sulfuretin, and cudratricusxanthone A, exerted anti-inflammatory effects via the induction of HO-1 expression [47,48,49,50]. Hence, our data suggest that EARDP exhibits antioxidant and anti-inflammatory effects in LPS-stimulated RAW 264.7 cells, via the regulation of Nrf2/HO-1 signaling pathway, as well as the NF-κB pathway.

Pharmacological studies suggest that MAPK signaling cascades act as a major regulatory pathway in Nrf2 activation associated with the expression of HO-1 [51]. The MAPKs, which include ERK, p38 MAPK, and JNK as three major members, play a critical role in modulating inflammatory responses as important mediators of intracellular signal transduction [52]. The serine–threonine kinase, Akt, is a phosphorylation-activated downstream kinase of PI3K. The phosphorylation of MAPKs and Akt plays an important role in LPS-induced macrophage activation, by inducing the regulation of NF-κB signaling [53]. Our results revealed that EARDP clearly inhibited the phosphorylation of p38 MAPK, ERK, JNK, and Akt in RAW 264.7 macrophages stimulated by LPS. In order to further establish that the inhibition of inflammatory signaling molecules by EARDP was related to the down-regulation of MAPKs and the Akt signaling pathway, we evaluated the effect of specific inhibitors of MAPKs and Akt on LPS-induced NO generation. Interestingly, the treatment with EARDP alone effectively suppressed the LPS-induced NO production in RAW 264.7 macrophages. In EARDP-treated RAW264.7 macrophages, however, LPS-induced NO production was not significantly suppressed by the specific inhibitors, including PD98059 (inhibitor of ERK), SB203580 (inhibitor of p38), and LY294002 (inhibitor of Akt). In addition, for unknown reasons, the ERK inhibitor PD98059 partially abrogated the inhibitory effect of EARDP. Taken together, our results suggest that the anti-inflammatory effects of EARDP were related to the inactivation of MAPKs and Akt signaling pathways, by suppressing the phosphorylation of MAPKs and Akt, which may control the downregulation of inflammatory mediators and cytokines in LPS-stimulated RAW 264.7 macrophages. A previous study reported that the aqueous extract of *Achyranthes japonica* roots, a medicinal herb, showed the inhibitory effects on iNOS expression and NF-κB activation via the inactivation of ERK, JNK and p38 in LPS-activated RAW 264.7 macrophages [54]. It has also been reported that aqueous extract of *Dipsacus asperoides* showed antioxidant and anti-inflammatory properties, through the suppression of the NF-κB and ERK1/2 pathway, but not the JNK or p38 MAPK pathways, in macrophages stimulated by LPS [55]. On the other hand, a previous study demonstrated that sea buckthorn downregulated the mRNA expression of pro-inflammatory cytokines and inhibited the phosphorylation of the p38 and SAPK/JNK MAPK pathways in LPS-stimulated RAW264.7 macrophages. These anti-inflammatory activities of the extracts may be likely to be related to the bioactive components, including polyphenolic compounds and flavonoids [56].

Furthermore, we investigated the effects of EARDP against TPA-induced edema in mouse ear. We found that the increased ear thickness and the synthesis of TNF-α and IL-1β induced by TPA were significantly attenuated by treatment with EARDP. In our previous study, we demonstrated that aged black garlic possessing strong antioxidant activity markedly inhibited TPA-induced dermatitis in a mouse model [57]. Similarly, other studies reported that specific medicinal plant extracts and their bioactive compounds play a protective role in inflammation induced by TPA [58,59,60]. These results indicate that EARDP exhibits in vivo anti-inflammatory properties against TPA-induced skin inflammation. These findings suggest that the protective effects of EARDP against oxidative stress and inflammation are possibly mediated via the regulation of Nrf2/HO-1 and NF-κB signaling pathways in LPS-activated RAW 264.7 macrophages, as well as the in vivo animal model induced by TPA.

## 5. Conclusions

In conclusion, EARDP attenuates the intracellular ROS and MDA levels, as well as inflammatory responses, by up-regulating HO-1 expression via the Nrf2 signaling pathway, inhibition of NF-κB activation and suppression of MAPK signaling pathway, leading to the decreased production of pro-inflammatory mediators and cytokines in LPS-stimulated RAW 264.7 macrophages. Further, EARDP suppressed TPA-induced ear edema and the synthesis of pro-inflammatory cytokines in a mouse model, suggesting that EARDP represents a promising natural compound protective against oxidative inflammatory diseases. Thus, treatment with EARDP enriched phenolic compounds and flavonoids can be used to protect against inflammatory disease. The present study may represent the beginning of the refinement and use of EARDP as preventive or curative natural substances in LPS-induced inflammatory diseases.
